# Whey-Derived Peptides Interactions with ACE by Molecular Docking as a Potential Predictive Tool of Natural ACE Inhibitors

**DOI:** 10.3390/ijms21030864

**Published:** 2020-01-29

**Authors:** Yara Chamata, Kimberly A. Watson, Paula Jauregi

**Affiliations:** 1 Harry Nursten Building, Department of Food and Nutritional Sciences, University of Reading, Reading RG6 6AP, UK; 2 Harborne Building, School of Biological Sciences, University of Reading, Reading RG6 6AP, UK

**Keywords:** ACE-inhibitory activity, whey peptides, molecular docking, hypertension

## Abstract

Several milk/whey derived peptides possess high in vitro angiotensin I-converting enzyme (ACE) inhibitory activity. However, in some cases, poor correlation between the in vitro ACE inhibitory activity and the in vivo antihypertensive activity has been observed. The aim of this study is to gain insight into the structure-activity relationship of peptide sequences present in whey/milk protein hydrolysates with high ACE inhibitory activity, which could lead to a better understanding and prediction of their in vivo antihypertensive activity. The potential interactions between peptides produced from whey proteins, previously reported as high ACE inhibitors such as IPP, LIVTQ, IIAE, LVYPFP, and human ACE were assessed using a molecular docking approach. The results show that peptides IIAE, LIVTQ, and LVYPFP formed strong H bonds with the amino acids Gln 259, His 331, and Thr 358 in the active site of the human ACE. Interestingly, the same residues were found to form strong hydrogen bonds with the ACE inhibitory drug Sampatrilat. Furthermore, peptides IIAE and LVYPFP interacted with the amino acid residues Gln 259 and His 331, respectively, also in common with other ACE-inhibitory drugs such as Captopril, Lisinopril and Elanapril. Additionally, IIAE interacted with the amino acid residue Asp 140 in common with Lisinopril, and LIVTQ interacted with Ala 332 in common with both Lisinopril and Elanapril. The peptides produced naturally from whey by enzymatic hydrolysis interacted with residues of the human ACE in common with potent ACE-inhibitory drugs which suggests that these natural peptides may be potent ACE inhibitors.

## 1. Introduction

The number of people with unhealthy living habits who have developed cardiovascular disease (CVD) has increased in recent years. The WHO reported that an estimated 17.9 million people lose their lives as a result of cardiovascular disease every year [1]. CVDs have become the leading cause of death globally [2]. High blood pressure (hypertension) is one of the most important well-defined risk factors for CVD [3], therefore, cardiovascular diseases can be prevented with blood-pressure lowering treatment. Hypertension is regulated by the renin-angiotensin system (RAS), through modulating the angiotensin-converting enzyme ACE, bradykinin and other factors [4,5,6].

ACE (dipeptidyl carboxypeptidase, EC 3.4.15.1) is a zinc metallopeptidase, found in male genital, vascular endothelial, neuro-epithelial, and absorptive epithelial cells [7,8,9], and displays both endopeptidase and exopeptidase activities, acting on a wide range of substrates [10]. ACE is a key enzyme for regulating blood pressure in the renin-angiotensin system. Renin cleaves the N-terminal segment of angiotensinogen from the biologically inert AT-1. ACE then hydrolyzes AT-1 by cleaving the carboxyl terminal His-Leu dipeptide from the inactive AT-1 to the active angiotensin II (AT-2), a potent vasoconstrictor responsible for the development of hypertension [5,6,11,12]. ACE also indirectly influences the kallikrein–kinin system, by promoting the inactivation and degradation of the catalytic function of bradykinin, a vasodilator involved in blood pressure control [11,12,13]. By repressing AT-2 production and restraining bradykinin degradation, ACE inhibitory peptides control the increase of blood pressure [13].

Consequently, ACE-inhibiting natural products have been vigorously investigated during the last decades, due to their potential in lowering blood pressure during hypertension. Among various types of bioactive peptides, ACE-inhibitory peptides from food sources have been most extensively studied for their potential use as natural alternatives to drugs for reducing blood pressure through binding and inhibiting ACE, and thus preventing and managing hypertension [14,15]. Food-derived peptides are believed to represent a healthier and more natural alternative source for chronic treatment of hypertension. Moreover, and although the inhibitory capacity of food-derived peptides is lower than that of chemically-designed antihypertensive drugs, such as Captopril, Sampatrilat, Lisinopril, and Enalapril, it is thought that food-derived peptides are safer than pharmaceutical drugs due to their lack of some drug-associated adverse side effects such as angioedema, skin rashes, and dry cough [6,16]. However, considering the lack of consensus in their physiological antihypertensive effects in different human populations, the role of food peptides in regulating blood pressure is still a subject of ongoing debate [17,18,19].

Although different animal and plant proteins have been used in the development of functional foods providing antihypertensive activity, milk is the main source of antihypertensive ACE-inhibitory peptides reported to date [20]. Milk is made up of 3.5% proteins of which 80% are caseins, classified as α-, β- and k-caseins, and 20% whey proteins. Whey contains α-lactalbumin, β- lactoglobulin and other minor proteins. Upon the degradation of milk proteins, peptide fragments with many biological effects that can be different from those of the parent protein, are released. Several bioactive peptides in milk proteins have been identified [21], and they serve an array of biological activities, including angiotensin-converting enzyme (ACE) inhibition, antimicrobial, antioxidative functions, dipeptidyl peptidase IV (DPP-IV) inhibition, opioid agonist and antagonist activities, immunomodulation, and mineral binding [22]. Several milk/whey derived peptides possess high in vitro ACE inhibitory activity; particularly, hydrolysates of whey proteins, caseinates, fractions-enriched in individual milk proteins, and whole milk proteins have been reported to be a good source of ACE-inhibitory peptides [14]. Ile-Pro-Pro (IPP) has been identified as the most potent ACE inhibitor from milk protein, and it is derived from casein [23]. The antihypertensive activity of this tripeptide has been demonstrated in several animal studies and human trials [24]. However, in some cases, poor correlation between the in vitro ACE inhibitory activity of milk-derived peptides and the in vivo antihypertensive activity has been observed. This can be partly due to digestion which renders less active peptide sequences and/or due to their low bioavailability [25]. Also, antihypertensive activity may be exerted by mechanisms other than ACE inhibition [26,27], e.g., specific ACE inhibitors were demonstrated to increase the risk of microscopic colitis in a recent study, suggesting that milk-derived peptides may exert their antihypertensive activity through the microbiome [28]. 

The activity of these peptides depends on their inherent amino acid composition and sequence [29,30]. Shorter peptides up to 12 amino acids with hydrophobic and positively charged amino acids at the carboxyl end are more likely to interact with ACE [31]. In terms of favorable structure–function relationship for high ACE-inhibitory activity, dipeptides including bulky and hydrophobic amino acids are more potent whereas tripeptides having aromatic amino acids at the C-terminus end, positively charged amino acids in the middle and hydrophobic amino acids at the N-terminus end, are most potent [31]. Kobayashi et al. (2008) investigated the effects of aromatic amino acids in the third position of the tripeptides on ACE-inhibitory activity. They found that the difference in the ACE-inhibitory activity between the bioactive peptides (IKW, LKW, IKY, and LKF) resulted from the aromatic amino acids W, Y, and F. The highest inhibitory activity was presented by LKW, with the largest amino acid in the C-terminal. Accordingly, ACE-inhibitory activity is affected by the size of the amino acid, as well as its hydrophobicity [32]. In the same study, Kobayashi et al. (2008) examined the effects of the charged amino acid in the second position and they reported that in order to obtain a high inhibitory activity, it is essential to have a positively charged residue next to an aromatic residue. They also highlighted that the tripeptide sequence consisting of either I or L + positively charged amino acids + aromatic amino acids is likely to have high ACE-inhibitory activity. The charged amino acid takes part in binding by ACE while the bulky aromatic amino acid prevents the access between substrates and the active site of ACE [32]. Some studies have indicated that tri-peptides show higher ACE-inhibitory activity, and the C terminus end of the tri-peptides substantially affects binding to ACE. Hydrophobic amino acid residues or Proline residues at the carboxyl end are important for ACE inhibition and inhibitors containing these residues are resistant to digestion [33].

ACE inhibitors are commonly discovered using classic investigation techniques which include hydrolysis of proteins with different proteolytic enzymes, isolation and purification of peptides using chromatographic systems, and synthesis of corresponding peptides for the confirmation of activity and structure [8]. Although some structure-activity relationships have been established for food protein derived peptides, they are still quite generic and thus could not be solely used to predict the ACE inhibitory activity of peptide sequences [34]. In order to avoid some challenges of the classical approach, such as having to apply cumbersome purification processes to isolate active peptides, the computer-based approach is considered a useful and effective method to identify novel peptides [35]. A number of docking algorithms are being used in multiple studies to predict potent ACE inhibitory peptides encrypted in food proteins [36,37,38,39,40,41,42], and more specifically in milk proteins [43,44], whereby attempts to understand the interactions between receptor and ligand are being attempted [45]. Molecular docking enables the investigation of the specific interactions between certain peptide sequences and specific binding site residues in ACE, which could help to provide a better prediction of bioactivity in vivo through a molecular understanding of the structure-function relationship. Such an approach can be a powerful tool that can be used in pre-screening potentially bioactive peptides, prior to their testing in vivo.

Herein we investigate the potential interactions between whey protein derived peptides with high ACE inhibitory activity and human ACE, utilising a molecular docking approach. This study follows our previous work [30], where the peptides were produced by enzymatic hydrolysis of whey and were further fractionated for their chemical and activity characterization. Moreover, the interactions between these peptides and ACE are compared with those of the ACE inhibitory drugs Sampatrilat, Captopril, Lisinopril, and Enalapril.

## 2. Results

### 2.1. Molecular Homology between Human ACE and Rabbit ACE

**Figure 1:**
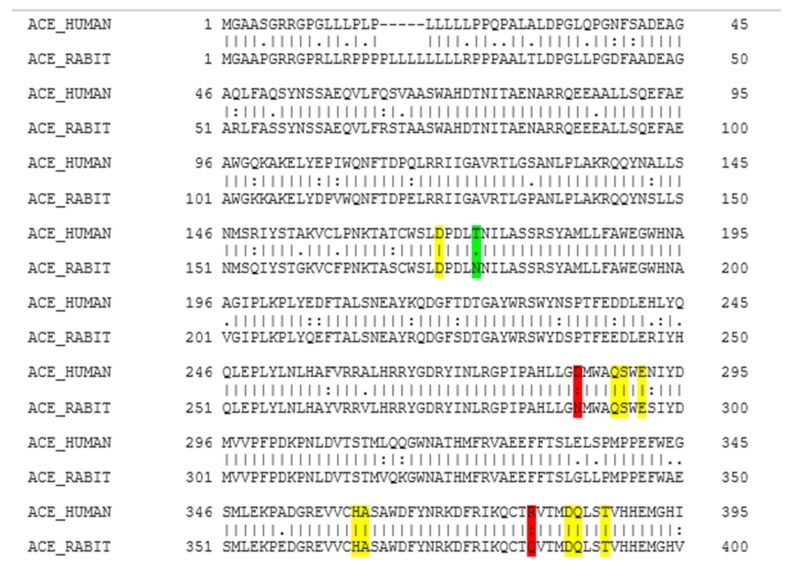
EMBOSS NEEDLE multiple sequence alignment results. Colour coding is as follows: Yellow indicates identical residues at the active site, green indicates similar residues at the active site, and red indicates that a part of the residue is similar. (I) residues are identical; (.) conserved change; (:) part of the residue is similar but not that conserved.

According to the EMBOSS NEEDLE results (Figure 1), there is 93.4% of similarity between human ACE and rabbit ACE. As shown in Figure 1, the structural comparison of these two enzymes indicates that there is a close homology between the human ACE and the rabbit ACE, and that the active sites between human ACE and rabbit ACE are very similar. The rabbit ACE is generally used for the in vitro testing of ACE inhibition, hence it can be assumed that similar results will be obtained with human ACE. There have not been any previous studies that reported the homology between the human ACE and the rabbit ACE. In a study by Soubrier et al. (1988), amino-terminal sequence analysis was conducted between amino-terminal amino acid sequences of human ACE and other mammalians (rabbit, calf, pig, and mouse), and a high degree of similarity was found between human ACE and these mammalians [46]. 

### 2.2. Molecular Docking

Molecular docking was conducted to elucidate the potential molecular interactions between the whey-protein derived peptide sequences and specific amino acids at the binding site of human ACE. The peptide sequences were docked into the binding site of the human ACE, using the X ray crystallographic structure of the human ACE receptor (PDB code 6F9V). The extracted co-crystallized ligand, Sampatrilat, [47] was first re-docked into the prepared protein to be used for docking in order to validate the docking procedure. The RMSD between the docked conformation, as generated by the program PyMol, and the native co-crystallized ligand conformation was 0.1 Å, which was well within the 2 Å grid spacing used in the docking procedure, demonstrating that the docking method to be used was valid and reliable. Additionally, the interactions between the docked ligand and the prepared target receptor mimicked those observed in the crystal structure of the same protein. 

**Figure 2:**
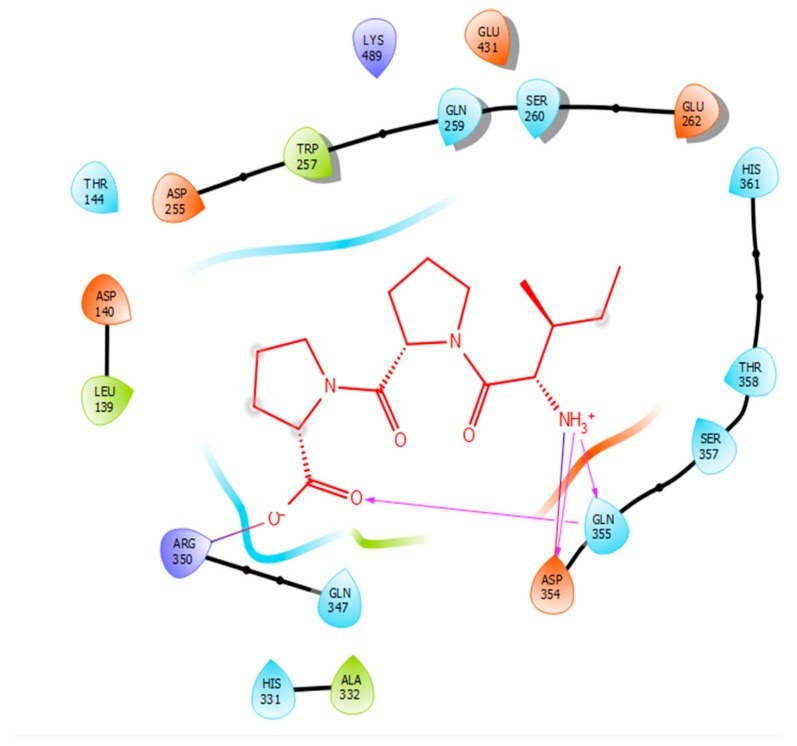
Docking results of the peptide IPP in the active site of human angiotensin I-converting enzyme (ACE). IPP is represented in red, interactions of human ACE residues with the peptide are indicated by arrows of different colours, with purple representing hydrogen bond interactions and blue arrows representing salt bridge interactions. The figure was generated using the software Maestro.

**Figure 3:**
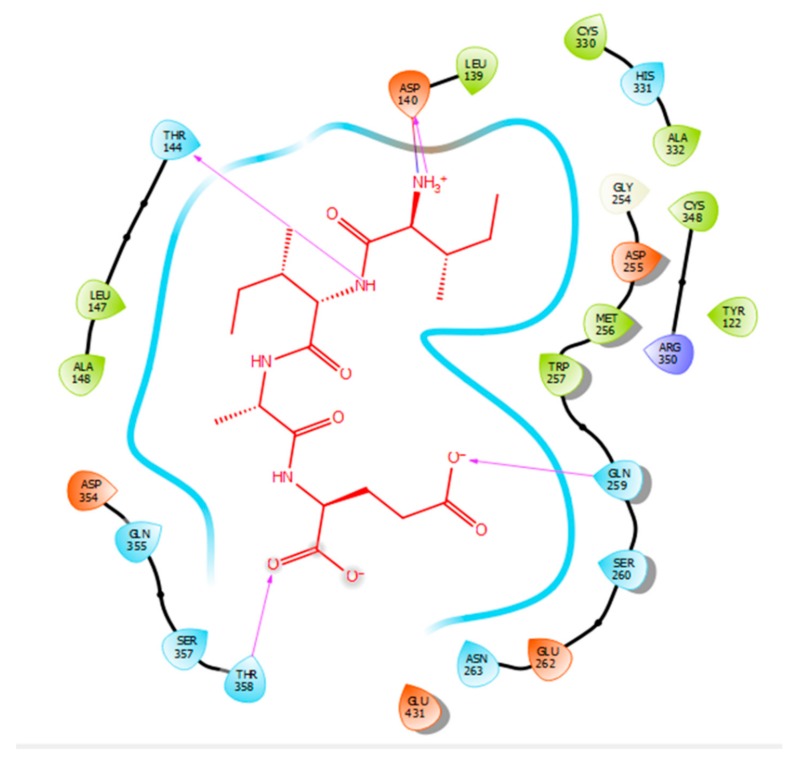
Docking results of the peptide IIAE in the active site human ACE. IIAE is represented in red, the interactions of human ACE residues with the peptide are indicated by arrows of different colours with purple representing hydrogen bond interactions, and blue arrows representing salt bridge interactions. The Figure was obtained using the software Maestro.

**Figure 4:**
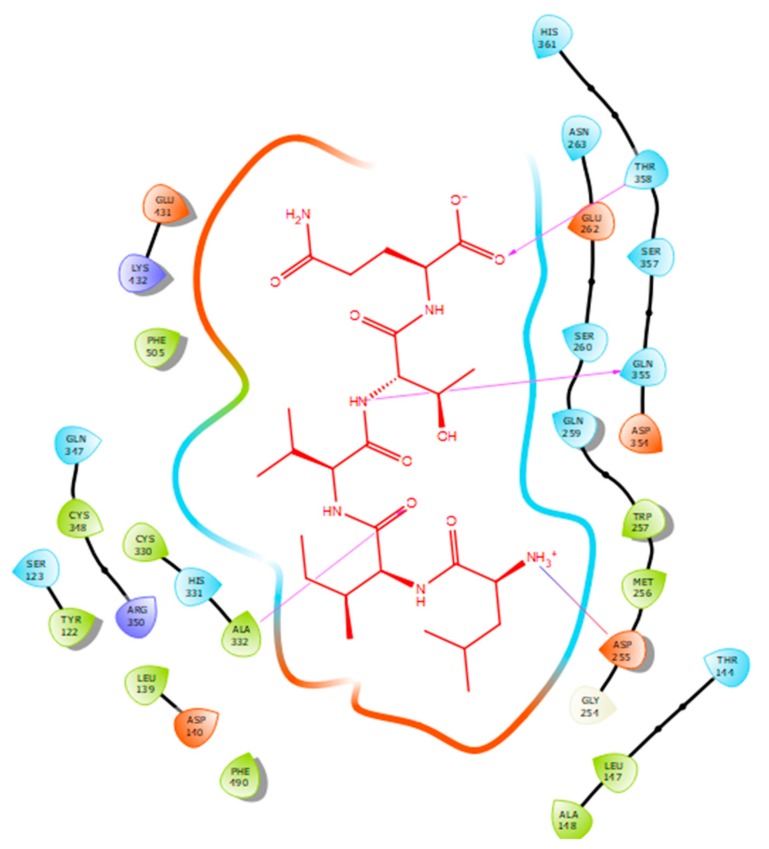
Docking results of the peptide LIVTQ in the human ACE active site. LIVTQ is represented in red, the interactions of human ACE residues with the peptide are indicated by arrows of different colours with purple representing hydrogen bond interactions, and blue arrows representing salt bridge interactions. The software Maestro was used for the generation of this figure.

**Figure 5:**
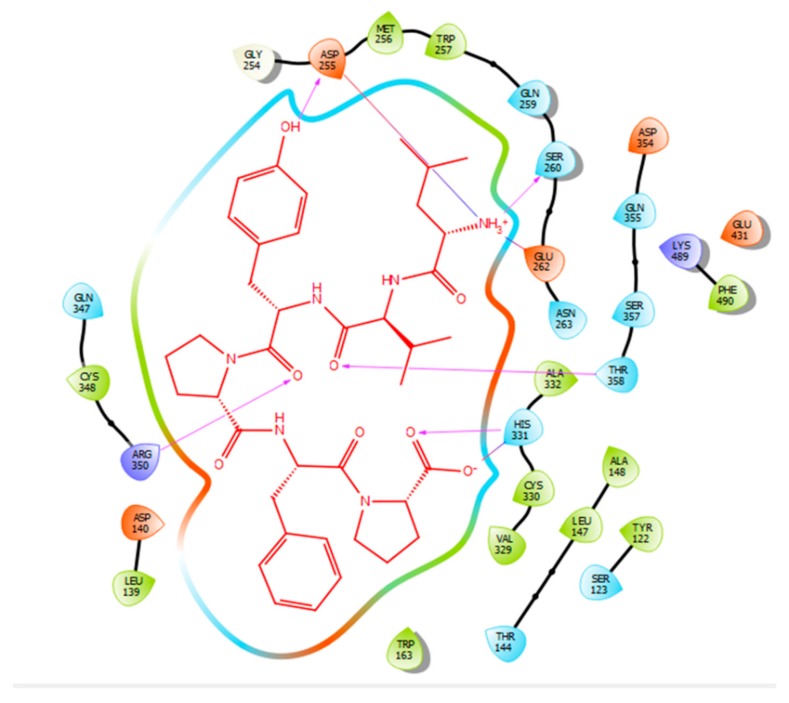
Docking results of the peptide LVYPFP in the human ACE active site. LVYPFP is represented in red, the interactions of human ACE residues with the peptide are indicated by arrows of different colours with purple representing hydrogen bond interactions, and blue arrows representing salt bridge interactions. The software Maestro was used for the generation of this figure.

Hydrogen bonds are a significant factor that contribute to the specificity and stability of protein-ligand interactions. Figure 2, Figure 3, Figure 4 and Figure 5 and Table 1, Table 2, Table 3 and Table 4 show the hydrogen bond interactions associated with each ligand and the surrounding ACE residues. IPP formed 3 hydrogen bonds with the ACE residues: one with Asp 354 and two with Gln 355 (Figure 2, Table 1). IIAE formed three hydrogen bonds with residues Thr 144, Gln 259, and Thr 358 (Figure 3, Table 2). LIVTQ formed three hydrogen bonds: one with Ala 332, one with Gln 355, and one with Thr 358 (Figure 4, Table 3). As for the ligand LVYPFP, five hydrogen bonds were formed with residues Asp 255, Ser 260, His 331, Arg 350, and Thr 358 (Figure 5, Table 4). It is interesting to note that several peptides had same H bonds in common: Thr 358 formed H bonds with three of the peptides, IIAE, LIVTQ, LVYPFP; Gln 355 with IPP and LIVTQ. Additionally, all except one of the aminoacids in the active site of the ACE were polar (charged and non charged) and some of these charged aminoacids were also involved in salt bridge (electrostatic) interactions (Table 1, Table 2, Table 3 and Table 4). IPP formed one salt-bridge interaction with residue Arg 350 (Figure 2, Table 1), whereas ligands IIAE and LIVTQ formed only one salt bridge interaction with residues Asp 140, and Asp 255, respectively (Figure 3 and Figure 4, Table 2 and Table 3). As for LVYPFP, two salt-bridge interactions were formed with residues Glu 262, and His 331 (Figure 5, Table 4). Both, LVYPFP and LIVTQ interacted with Asp 255 via H-bonding and a salt bridge, respectively; and, both IPP and LVYPFP interacted with Arg 350 via a salt bridge and H-bonding, respectively.

## 3. Discussion

Hydrogen bonds interactions were demonstrated to play a crucial role in stabilizing the docked ligand complexes [48]. The distance of hydrogen bond interactions between the whey derived peptides and ACE amino acid residues typically were short (< 3.0Å; Table 1, Table 2, Table 3 and Table 4), indicating that the peptides’ binding affinity to ACE was strong [49]. In addition, these peptides formed a number of favorable salt bridge interactions with ACE residues, indicating that the ligands can pack tightly into the binding site and effectively inhibit ACE. Furthermore, it is interesting to note that hydrophobic amino acid residues such as proline, leucine, and isoleucine were mainly involved in establishing strong interactions with ACE, which goes in accordance with what is reported in SAR (Table 1, Table 2, Table 3 and Table 4) [33]. 

**Figure 6:**
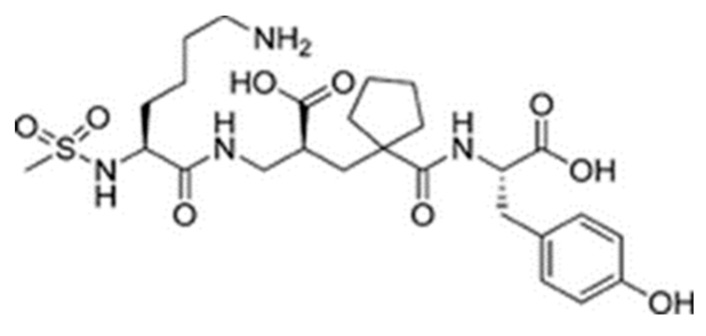
Chemical structure of Sampatrilat [47].

Sampatrilat ((S, S, S)-*N*-{1-[2-carboxy-3-(N-mesyllysylamino) propyl]-1-cyclopentylcarbonyl} tyr-osine) (Figure 6) is a potent dual inhibitor of ACE and neutral endopeptidase. In the treatment of chronic heart failure, Sampatrilat could potentially provide a greater benefit than traditional ACE inhibitors [50,51]. In a recent study investigating the binding of Sampatrilat to the active site of ACE, the amino acid residues involved in the interactions with Sampatrilat were reported [47]. Interestingly, IIAE, LIVTQ, and LVYPFP interacted with three of these previously identified amino acid residues: IIAE interacted with residue Gln 259, LVYPFP interacted with residue His 331 and IIAE, LIVTQ, and LVYPFP interacted with residue Thr 358. Furthermore, previous studies stated that the ACE-inhibitory drugs Captopril, Lisinopril, and Enalapril interact with ACE amino acid residues Gln281, His353, Glu384, Lys511, His 513, and Tyr520 [52,53,54]. Apart from the amino acid residues in common, Lisinopril was reported to interact with Ala 354, Tyr 523, and Glu 162, and Enalapril to interact with Ala 354 and Tyr 523 [52,54]. According to the docking results, the peptides IIAE and LIVTQ interacted also with two of these residues: IIAE interacted with residue Asp 140 in common with Lisinopril, and LIVTQ interacted with Ala 332 in common with Lisinopril and Enalapril. (Amino acids residues are reported according to the Sampatrilat (PDB code 6F9V) amino acid sequence numbering, please see Table A1). 

Overall, the docking results together with comparisons with the ACE inhibitory drugs provide strong evidence for the ACE inhibitory activity of IIAE, LIVTQ, and LVYPFP. In our previous work [31], IIAE, LIVTQ, and LVYPFP were identified as major peptides within fractions of high ACE inhibitory activity. Additionally, based on known structure-activity relationships, it was assumed that these were the main contributors to the ACE inhibitory activity measured. The docking results herein corroborate these assumptions and suggest that most probably these are potent ACE inhibitors that will contribute to the ACE inhibitory and antihypertensive activity in vivo. Further work will be needed, using pure synthesized peptides, to confirm ACE inhibition and activity in vivo.

## 4. Materials and Methods

### 4.1. Whey-Protein Derived Peptides

In our previous work where we characterized angiotensin-converting enzyme (ACE) inhibitory peptides produced by enzymatic hydrolysis of whey proteins [31], peptide sequences were identified as major peptides in fractions from the enzymatic hydrolysates CDP (casein-derived peptides) and β-lactoglobulin. The well-known antihypertensive peptide IPP, along with some other novel peptide sequences that have structural similarities with reported ACE inhibitory peptides, such as Leu-Val-Tyr-Pro-Phe-Pro (LVYPFP), Leu-Ile-Val-Thr-Gln (LIVTQ), and Ile-Ile-Ala-Glu (IIAE) were characterized and identified by a combination of chemical characterization (LC/MS; MS/MS) and SAR data. Their ACE inhibitory activity is summarized in Table 5; the IC_50_ is defined as the peptide concentration required to reduce the ACE activity by half. 

### 4.2. Homology between Human ACE and Rabbit ACE

EMBL-EBI (https://www.ebi.ac.uk/) was queried for human and rabbit ACE amino acid sequences, together with known three-dimensional protein structures. Reviewed sequences were selected and the protein sequence files were downloaded. The accession codes for the human ACE and the rabbit ACE used in this work are P12821 and P12822, respectively. The two sequences were then uploaded to Emboss Needle (https://www.ebi.ac.uk/Tools/psa/emboss_needle/) for multiple sequence alignment and comparison.

### 4.3. Molecular Docking

#### 4.3.1. Docking Validation

In order to validate the accuracy and the reliability of the docking procedure to be used in this study, the original ligand (extracted from the coordinate files and taken from the Protein Data Bank; PDB code 6F9V was docked into the corresponding crystal structure of the receptor, using the automated docking procedure in the program Surflex-Dock (SFXC) [56], as provided by SYBYL-X2.1. The docked ligand mode and orientation from the docking procedure were compared to that found in the actual crystal structure of the complex using Pymol and PDBeFold [57,58]. Following the docking procedure, the root mean square deviation (RMSD) between the docked ligand and the ligand, as found in the crystal structure, was calculated. The success of the docking process depended on whether the value of RMSD between the real and best-scored docked conformations were within the 2 Å grid spacing, used in the docking procedure [59], and whether the molecular interactions were replicated. In this case, Sampatrilat was docked into the human ACE receptor as validation of the docking procedure.

#### 4.3.2. Docking Procedure

Whey protein-derived peptides Ile-Pro-Pro (IPP), Leu-Ile-Val-Thr-Gln (LIVTQ), Ile-Ile-Ala-Glu (IIAE), and Leu-Val-Tyr-Pro-Phe-Pro (LVYPFP) were used as ligands in separate docking runs. Docking was performed using the docking algorithm Surflex-Dock, as provided in Sybyl-X 2.1. The X-ray crystallographic structure of sampatrilat-Asp in complex with Angiotensin-I-converting enzyme (PDB code 6F9V, 1.69 Å resolution) retrieved from the protein data bank (PDB) was chosen as the target protein for the docking studies, based on its high resolution structure co-crystallized with sampatrilat-Asp [47].

The Biopolymer Structure Preparation Tool, with the implemented default settings provided in the SYBYL programme suite, was used to prepare the protein structure for docking; hydrogens were added to the protein structure in idealised geometries, backbone and sidechains were repaired, residues were protonated, sidechain amides and sidechain bumps were fixed, stage minimization was performed, and all water and any ligand molecules were removed. 

The three-dimensional (3D) structure of each ligand was constructed, using the “Build Protein” tool, as provided in Sybyl-X. Once constructed, charges were assigned to each atom of each molecule, using Merck Molecular Force Field (MMFF94) charges. Localized energy minimizations were then performed, and the final structure for each ligand in its lowest energy conformation was used for subsequent docking experiments. The resulting 3D coordinate files were converted to a MOL2 format for subsequent use in Surflex-Dock experiments, as provided in the SYBYL-X 2.0 software suite.

Surflex-Dock is a search algorithm that utilizes an empirically derived scoring function whose parameters are based on protein-ligand complexes of known affinities and structures. This method employs a “protomol”, which is an idealized active site, as a target to generate presumed poses of molecules or molecular fragments. The protomol is employed as a mimic of the ideal interactions made by a perfect ligand to the active site of the protein. This molecular-similarity based alignment allows for optimization of potentially favorable molecular interactions, such as those defined by van der Waals forces and hydrogen bonds. In the present work, the protomol was defined by optimizing the threshold and bloat values to 0.5 and 0, respectively, to create a protomol that adequately described the binding pocket of interest. The extent of the protomol and its degree of coverage of an active site are controlled by these two parameters: the threshold value indicates the amount of buried-ness for the primary volume used to generate the protomol, and the bloat parameter determines the number of Ångstroms by which the search grid beyond that primary volume should be expanded. It is generally better to err on the side of a small protomol than on a protomol that is too large [60]. All parameters within the docking suite were left as the default values as established by the software [61,62]. Each peptide was then individually docked into the protomol site, using the “Docking Suite” application, as provided in the SYBYL programme suite. The docking results were visualised using the programme Maestro.

Molecular interactions, for the docking results, are reported according to the Sampatrilat (PDB code 6F9V) amino acid sequence numbering; for comparisons between different sequence numbering in studies referred here (See Appendix A (Table A1)). The software Maestro was used for the identification and characterisation of hydrogen bonds and salt-bridge interactions established between residues at the ACE active site and the peptides. 

## 5. Conclusions

For the first time, reported herein, potential interactions between the naturally produced peptides from whey and ACE have been investigated, using a molecular docking approach. Peptides, IPP, IIAE, LIVTQ, and LVYPFP formed strong H bonds and salt bridge interactions with residues in the active site of human ACE. Moreover, a comparison with commercial ACE inhibitory drugs showed that the natural peptides interacted similarly to the drugs mimicking the same interactions with ACE active site residues. This study provides strong evidence for the ACE inhibitory activity of milk derived peptides, which have not been tested in vivo before. The results of this study, of novel milk derived whey peptides, could lead to the production of novel ACE inhibitors.

## Figures and Tables

**Table 1:** IPP docking results.

Protein 69FV	Ligand IPP		
Residue	Atom Name	Interaction Type	Distance (Å)
NH_2_ Arg 350	O^−^ (Pro)	Salt bridge	2.86
OD2 Asp 354	NH^3+^ (Ile)	Hydrogen bond	1.86
OE1 Gln 355	NH^3+^ (Ile)	Hydrogen bond	1.81
NE2 Gln 355	O- (Pro)	Hydrogen bond	2.04

**Table 2:** IIAE docking results.

Protein 69FV	Ligand IIAE		
Residue	Atom Name	Interaction Type	Distance (Å)
OD2 Asp 140	NH (Ile)	Salt bridge	2.57
OG1 Thr 144	N (Ile)	Hydrogen bond	2.15
NE2 Gln 259	O (Glu)	Hydrogen bond	2.2
OG1 Thr 358	O (Glu)	Hydrogen bond	1.88

**Table 3:** LIVTQ docking results.

Protein 69FV	Ligand LIVTQ		
Residue	Atom Name	Interaction Type	Distance (Å)
OD2 Asp 255	NH3+ (Leu)	Salt bridge	4.79
N Ala 332	O (Ile)	Hydrogen bond	2.56
OE1 Gln 355	N (Thr)	Hydrogen bond	1.85
OG1 Thr 358	O- (Gln)	Hydrogen bond	1.88

**Table 4:** LVYPFP docking results.

Protein 69FV	Ligand LVYPFP		
Residue	Atom Name	Interaction Type	Distance (Å)
OD2 Asp 255	OH (Tyr)	Hydrogen bond	2.12
OG Ser 260	NH3+ (Leu)	Hydrogen bond	1.89
OE2 Glu 262	NH3+ (Leu)	Salt bridge	3.58
ND1 His 331	O (Pro)	Hydrogen bond	1.92
ND1 His 331	O (Pro)	Salt bridge	2.71
NH2 Arg 350	O (Tyr)	Hydrogen bond	2.61
OG1 Thr 358	O (Valine)	Hydrogen bond	1.75

**Table 5:** IC_50_ (μg/mL) value of the ACE-inhibitory peptide sequences.

Peptide	Protein Source	IC50 (μg/mL)	Reference
IPP	k-Casein	1.23	[23]
IIAE	β-Lg	128 *	[31]
LVYPFP	Casein	97	[55]
LIVTQ	β-Lg	113	[31]

* IC_50_ of β-Lg hydrolysate containing this peptide as one of the major peptides.

## Appendix A

**Table A1:** Comparisons between Captopril (PDB code 1O86) and Sampatrilat (PDB code 6F9V) sequence numbering.

Captopril (PDB Code 1O86) Amino Acid Sequence Numbering [52]	Sampatrilat (PDB Code 6F9V) Amino Acid Sequence Numbering [47]
Glu 162	Asp 140
Gln 281	Gln 259
His 353	His 331
Ala 354	Ala 332
